# Identification of genetic variation that determines human trehalase activity and its association with type 2 diabetes

**DOI:** 10.1007/s00439-013-1278-3

**Published:** 2013-03-07

**Authors:** Yunhua L. Muller, Robert L. Hanson, William C. Knowler, Jamie Fleming, Jayita Goswami, Ke Huang, Michael Traurig, Jeff Sutherland, Chris Wiedrich, Kim Wiedrich, Darin Mahkee, Vicky Ossowski, Sayuko Kobes, Clifton Bogardus, Leslie J. Baier

**Affiliations:** Diabetes Molecular Genetics Section, Phoenix Epidemiology and Clinical Research Branch, National Institute of Diabetes and Digestive and Kidney Disease, National Institutes of Health, 455 North 5th Street, Phoenix, AZ 85004 USA

## Abstract

A prior linkage scan in Pima Indians identified a putative locus for type two diabetes (T2D) and body mass index (BMI) on chromosome 11q23-25. Association mapping across this region identified single nucleotide polymorphisms (SNPs) in the trehalase gene (*TREH*) that were associated with T2D. To assess the putative connection between trehalase activity and T2D, we performed a linkage study for trehalase activity in 570 Pima Indians who had measures of trehalase activity. Strong evidence of linkage of plasma trehalase activity (LOD = 7.0) was observed in the *TREH* locus. Four tag SNPs in *TREH* were genotyped in these subjects and plasma trehalase activity was highly associated with three SNPs: rs2276064, rs117619140 and rs558907 (*p* = 2.2 × 10^−11^–1.4 × 10^−23^), and the fourth SNP, rs10790256, was associated conditionally on these three (*p* = 2.9 × 10^−7^). Together, the four tag SNPs explained 51 % of the variance in plasma trehalase activity and 79 % of the variance attributed to the linked locus. These four tag SNPs were further genotyped in 828 subjects used for association mapping of T2D, and rs558907 was associated with T2D (odds ratio (OR) 1.94, *p* = 0.002). To assess replication of the T2D association, all four tag SNPs were additionally genotyped in two non-overlapping samples of Native Americans. Rs558907 was reproducibly associated with T2D in 2,942 full-heritage Pima Indians (OR 1.27 *p* = 0.03) and 3,897 “mixed” heritage Native Americans (OR 1.21, *p* = 0.03), and the strongest evidence for association came from combining all samples (OR 1.27 *p* = 1.6 × 10^−4^, *n* = 7,667). However, among 320 longitudinally studied subjects, measures of trehalase activity from a non-diabetic exam did not predict those who would eventually develop diabetes versus those who would remain non-diabetic (hazard ratio 0.94 per SD of trehalase activity, *p* = 0.29). We conclude that variants in *TREH* control trehalase activity, and although one of these variants is also reproducibly associated with T2D, it is likely that the effect of the SNP on risk of T2D occurs by a mechanism different than affecting trehalase activity. Alternatively, *TREH* variants may be tagging a nearby T2D locus.

**Electronic supplementary material:**

The online version of this article (doi:10.1007/s00439-013-1278-3) contains supplementary material, which is available to authorized users.

## Introduction

A prior genomic linkage scan in Pima Indians identified an obesity susceptibility locus on chromosome 11q23-25 (LOD = 3.6). There was also suggestive evidence that the same genomic region contains a susceptibility locus for type two diabetes (T2D, LOD = 1.7) (Hanson et al. 1998). This region of linkage to obesity and/or T2D was replicated in several other studies including Caucasians who are part of the Framingham study (Atwood et al. 2002), part of the Breda Study Cohort (van Tilburg et al. 2003), Utah Caucasians (Elbein et al. 1999) and Mexican-Americans who are part of the GENNID study (Duggirala et al. 2003). However, no variant in this region on chromosome 11 was reported to be among the top associations in large-scale meta-analyses of genome-wide association data for T2D or obesity in Caucasians (Zeggini et al. 2008; Morris et al. 2012; Willer et al. 2009).

The region of linkage in Pima Indians spans approximately 23 Mb, from approximately 112–134 Mb and contains ~345 genes. This segment of chromosome 11 contains the dopamine receptor D2 gene, which is a good physiologic candidate gene for obesity; however, variations in this gene do not account for the linkage signal for BMI in Pima Indians (Jenkinson et al. 2000). In subsequent association mapping studies, several SNPs with nominally significant associations with T2D are mapped to or near *TREH, UBASH3B, KIRREL3* and *SNX19.* Some of the most strongly associated variants are in or near the potential candidate gene *TREH* (Hanson et al. 2006), which codes for trehalase. In British and Nigerian studies, plasma trehalase activity was higher in diabetic subjects than non-diabetic subjects (Eze 1989). Higher serum trehalase activity has also been observed in diabetic subjects with glycosuria compared to diabetic subjects without glycosuria (Isichei and Gorecki 1993). In mice, trehalase activity is also elevated in both alloxan-induced and genetic (Ob/Ob, Db/Db) diabetic mice (Baumann et al. 1981; Ramaswamy and Flint 1980). However, the causal direction underlying this association is not known.

Our association mapping for the T2D locus on chromosome 11q23-25 led us to perform detailed analysis of the *TREH* gene structure and its enzymatic activity, reported herein. Trehalase splits the disaccharide α, α-trehalose into two molecules of glucose. This enzyme is found in bacteria, insects and mammals. In mammals, it is restricted to the small intestine, kidney, liver and bile (Kalf and Reider 1958; Kenny and Maroux 1982). In humans, the physiological function of trehalase is to digest dietary trehalose in the small intestine, but its exact role in carbohydrate metabolism is not clear.

One of the notable properties of substrate trehalose is its ability to protect cellular integrity during desiccation. Desert plants, bacteria, yeast, mushrooms and insects all manufacture trehalose as a defense mechanism against dehydration (Wingler 2002; Elbein et al. 2003). Trehalose can also protect against oxidative stress (Echigo et al. 2012). In humans, the main dietary sources of naturally occurring trehalose are mushrooms, baker and brewer’s yeast and certain kinds of shrimp. However, trehalose’s unique properties of protecting the integrity of a cell during desiccation have made it an important additive in the food and drug industries. It is also commonly used as a sweetener in bakery goods, beverages, confectionery, fruit jam, breakfast cereals, rice, and noodles (Richards et al. 2002).

Some individuals are unable to absorb trehalose, and the hereditary trehalose malabsorption is correlated with trehalase deficiency. In Greenland, the prevalence of trehalase deficiency has been reported to be 8 %, which is considerably higher than that seen elsewhere (Gudmand-Høyer et al. 1988). Recently, this sugar has been widely touted as an alternative to sucrose in diets proposed to prevent diabetes, due to reduced glycaemic and insulinaemic responses following trehalose ingestion (van Can et al. 2009). On account of this, and on account of the linkage studies described above, we sought to explore the relationship between trehalase activity and T2D and also determine the genetic basis for trehalase activity.

## Methods

### Subjects

All subjects in this study are participants of a longitudinal study of the etiology of T2D among the Gila River Indian Community in Arizona, where most of the residents are Pima Indians or Tohono O’odham (a closely related tribe, Knowler et al. 1978). Diabetic status was determined by an oral glucose tolerance test according to the criteria of the American Diabetes Association (The Expert Committee on the Diagnosis and Classification of Diabetes Mellitus 1997). The original observation of genetic linkage on chromosome 11q23-25 was determined in 966 individuals from 264 nuclear families who were selected for having family members with T2D (Hanson et al. 1998). Among these 966 individuals, 828 had complete data on covariates for association analysis with T2D, and, thus, were used for association mapping of the suggestive T2D linkage signal. Characteristics of these 828 individuals were: 648 (78 %) had T2D; 320 (39 %) were men; mean (±SD) BMI = 38.4 ± 8.5 kg/m^2^; and mean age at last exam =48.3 ± 13.0 years. Replication of SNP associations with T2D was assessed in two additional, non-overlapping samples from our longitudinal study on the Gila River Indian Community. The first replication sample included all additional individuals who were full-heritage Pima Indian (*n* = 2,942): 1,149 (39 %) had T2D; 1,294 (44 %) were men; mean BMI = 37.1 ± 8.6 kg/m^2^; and mean age = 37.1 ± 16.6 years. The second replication sample included all additional individuals who were not full-heritage Pima Indian (*n* = 3,897). Heritage in this “mixed heritage” group was, on average, ½ Pima and ¾ American Indian, which may include other tribes. Among these individuals, 727 (19 %) had T2D; 1,791 (46 %) were men; mean BMI = 34.6 ± 8.8 kg/m^2^; and mean age at the last exam = 27.6 ± 13.7 years.

### Sequencing of the trehalase gene and genotyping of SNPs

Sequencing of the *TREH* coding regions and putative promoter region was initially done in 39 Pima subjects using Big Dye terminator (Applied Biosystems) on an automated DNA capillary sequencer (model 3730; Applied Biosystems). These 39 subjects (68 % had T2D) were selected to maximize identification of genetic variation. All 39 subjects were from different nuclear Pima Indian families, and were selected as having the most diverse combinations of microsatellite markers near the *TREH* locus in our linkage study. More recently, complete exome sequencing was performed in 177 additional full-heritage Pima Indians (28 % had T2D), each from a different nuclear family, using next-generation sequencing technology (ShanghaiBio Corp., North Brunswick, NJ). In addition, whole genome sequence data are currently being generated (Complete Genomics Inc, Mountain View, CA); to date, genomic data on 30 Pima Indians (17 % had T2D) are available for analyses.

SNPs identified by sequencing and tag SNPs (in intron and flanking regions) selected from databases were genotyped for association analyses using the TaqMan Allelic Discrimination (AD) Assay (Applied Biosystems) on an ABI Prism 7700 (Applied Biosystems) or SNPlex genotyping System 48-plex (Applied Biosystems) on an automated DNA capillary sequencer (model 3730; Applied BioSystems).

### Measurement of plasma trehalase activity

Plasma samples for measurement of trehalase activity levels were available on 570 subjects who were part of the original linkage study. These samples were drawn during an exam where the subject was determined to be non-diabetic. Among these 570 subjects, 320 had follow-up information from subsequent exams, and 214 of the 320 subjects subsequently developed diabetes. Plasma trehalase activity was measured using the method adapted from Eze (1989). In brief, each plasma sample was incubated with or without substrate trehalose, and glucose concentration was measured by a glucose oxidase method (Beckman Instruments). The liberated glucose (i.e., the difference) was taken as trehalase enzyme activity. Each sample was measured in duplicate and the mean of the two measurements was used in statistical analyses.

### Statistical analysis

Statistical analyses were performed using the software of the SAS Institute (Cary, NC). Linkage analysis of plasma trehalase activity, as a quantitative trait, was conducted for sibships by means of variance-components methods (Amos et al. 1996). The GENEHUNTER program (Pratt et al. 2000) was used to derive multipoint estimates of the proportion of alleles identical by descent at each chromosomal location for these analyses. Linkage analysis of diabetes, accounting for the age-specific occurrence of the disease, was accomplished with a cumulative incidence “residual” method. It uses age and affection status to produce an “age-adjusted” diabetes score that can be analyzed as a quantitative trait (Hanson and Knowler 1998). Single trait analysis suggested that both trehalase activity and diabetes were linked to the same region; therefore, bivariate linkage analysis was conducted by covariance-components models (Lange and Boehnke 1983; Almasy et al. 1997) to assess the extent to which the presumed gene affects both traits. Detailed linkage analysis methods have been described previously (Hanson et al. 1998).

The general association of genotypes with T2D was assessed with logistic regression analysis and was adjusted for covariates (age, sex, birth year and heritage). The model was fit with a generalized estimating equation (GEE) technique to account for correlation among siblings. Genotype was analyzed as a numeric variable representing the number (0, 1, 2) of copies of a given allele (i.e., an “additive” model). Estimates of the proportion of European ancestry were derived by the method of Hanis et al. (1986) from 45 informative markers with large differences in allele frequency between populations (Tian et al. 2007) for use as a covariate in these analyses. The association of trehalase activity with genotype was assessed by a linear mixed model that incorporated a random effect to account for the correlation among siblings in addition to fixed effects for genotype, age and sex. *p* values were calculated by the likelihood ratio test. Linkage disequilibrium (LD), haplotype blocks and haplotype frequencies were analyzed by Haploview (version 4.2) (Gabriel et al. 2002).

The extent to which associations could explain the linkage signal was assessed by a model that tests the amount by which an associated polymorphism reduces the variance attributed to the linked locus (Hanson and Knowler 2008). This method fits a bivariate linkage model to the original trait and to the residual adjusted for genotype. To assess the potential independent contribution of multiple associated SNPs, conditional analyses were conducted in which a SNP of interest was added to a model containing one or more additional SNPs. These analyses were conducted in a “step-wise” fashion to identify a set of SNPs that were associated with the trait of interest. Haplotypes were analyzed by a modification of the zero recombinant haplotyping method as previously described (Vozarova de Courten et al. 2005). Briefly, the MLINK program is used to calculate the probability that each individual carries one or two copies of each haplotype, given their genotypes and the genotypes of their family members. These probabilities are then used to conduct the analysis for each haplotype in a fashion analogous to that for single SNP.

## Results

### Association mapping of the region of suggestive linkage to T2D

**Fig. 1:**
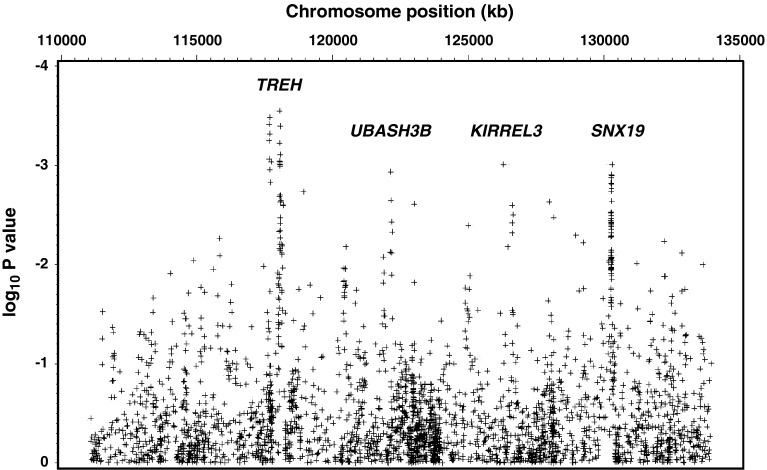
Association analyses with T2D for 2882 SNPs which span 23 Mb of chromosome 11 (position:111 Mb-134 Mb, NCBI B36) in 828 family-based full-heritage Pima subjects who participated in the linkage study. *p* values were adjusted for age and sex. Association with T2D was analyzed using a “model-based standard error” method. SNPs with a minor allele frequency <0.05 were omitted in the plots

To identify the variant(s) that gave rise to the suggestive linkage signal to T2D on chromosome 11q, 2882 SNPs were genotyped in 828 subjects and analyzed for association with T2D. These markers span 23 Mb of chromosome 11 (position 111 Mb- 134 Mb, NCBI build 36) with an inter-SNP distance of <10 Kb. Their associations with T2D (*p* values <10^−3^) are mapped to or near *TREH, UBASH3B, KIRREL3* and *SNX19.* (Fig. 1, *p* values <10^−3^, adjusted for age, sex and birth year). The strongest associations with T2D were clustered around 118 Mb which contains the *TREH* locus (adjusted *p* <0.002); therefore, SNP variation at this locus, and trehalase activity were further studied in Pima Indians.

### Linkage and association mapping of TREH activity

**Fig. 2:**
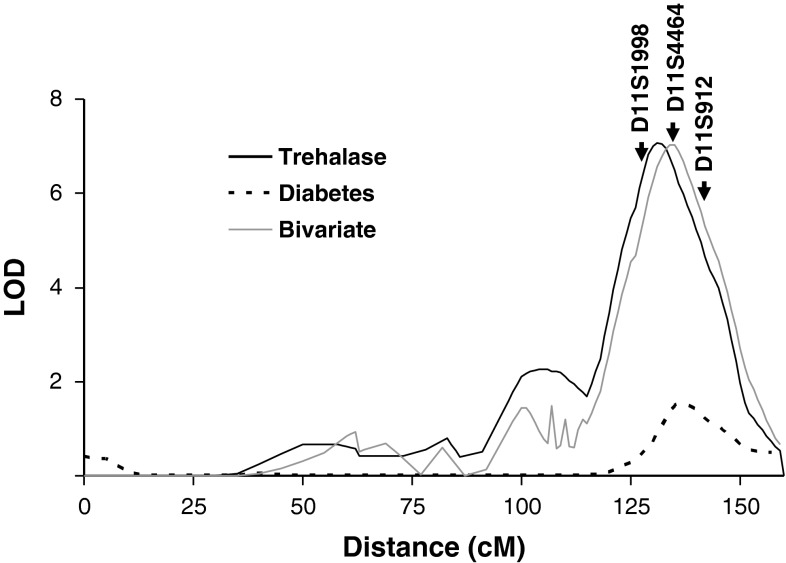
Multipoint results for linkages with plasma trehalase activity and T2D in/near the *TREH* locus on chromosome 11q23. Distances are from the *p*-terminal end of the chromosome, on the basis of a genetic map derived from data from the present study

Trehalase activity was measured in plasma samples from 570 Pima Indians who were determined to be non-diabetic when their blood was drawn. In these 570 subjects, linkage analysis showed strong linkage (LOD = 7.0) of plasma trehalase activity to markers on Chromosome 11q23 at 131 cM, near the *TREH* locus (Fig. 2). The linkage peak mapped in the vicinity of three microsatellite markers, D11S1998, D11S4464 and D11S912, which coincided with the suggestive linkage peak for age-adjusted T2D (LOD = 1.7) at 137 cM. Since both trehalase activity and T2D were linked to the same region, a multipoint bivariate analysis was conducted to assess the extent to which a single locus may affect both traits. Although bivariate analysis showed a LOD of 7.0, the estimated genetic correlation between the trehalase and T2D loci (*r* = −0.47) is not statistically significantly different from 0 (*p* = 0.09), as expected if linkage were coincidental, nor is it significantly different from −1 (*p* = 0.23), as expected with high pleiotropy. Thus, linkage analysis in the present set of families has limited power to resolve the extent of overlap between variants affecting trehalase levels and diabetes risk, and further association studies were conducted.

**Fig. 3:**
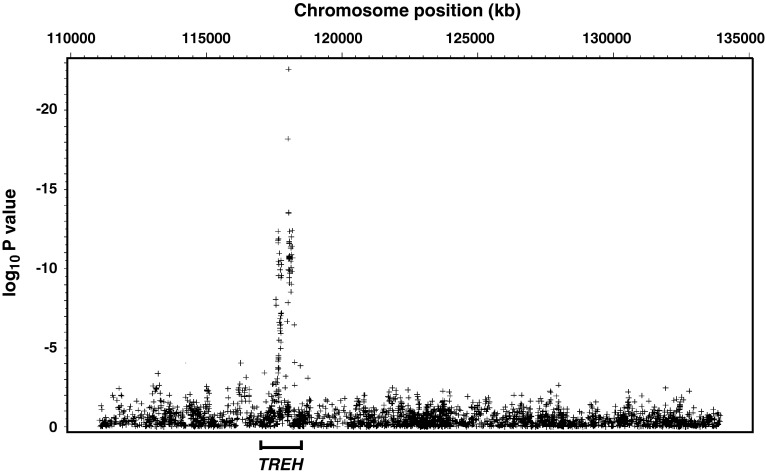
Association analyses with trehalase activity for 2882 SNPs which span 23 Mb of chromosome 11 (position:111 Mb-134 Mb, NCBI B36) in 570 non-diabetic Pima subjects who are part of the linkage study. *p* values were adjusted for age and sex. SNPs with a minor allele frequency <0.05 were omitted in the plots

The 2882 SNPs genotyped for association mapping of the T2D signal on chromosome 11 were analyzed for association with TREH activity. Multiple SNPs that mapped within the *TREH* locus had very strong associations with TREH activity (Fig. 3; *p* values adjusted for age and sex).

### Sequencing of *TREH* and selection of tag SNPs

**Fig. 4:**
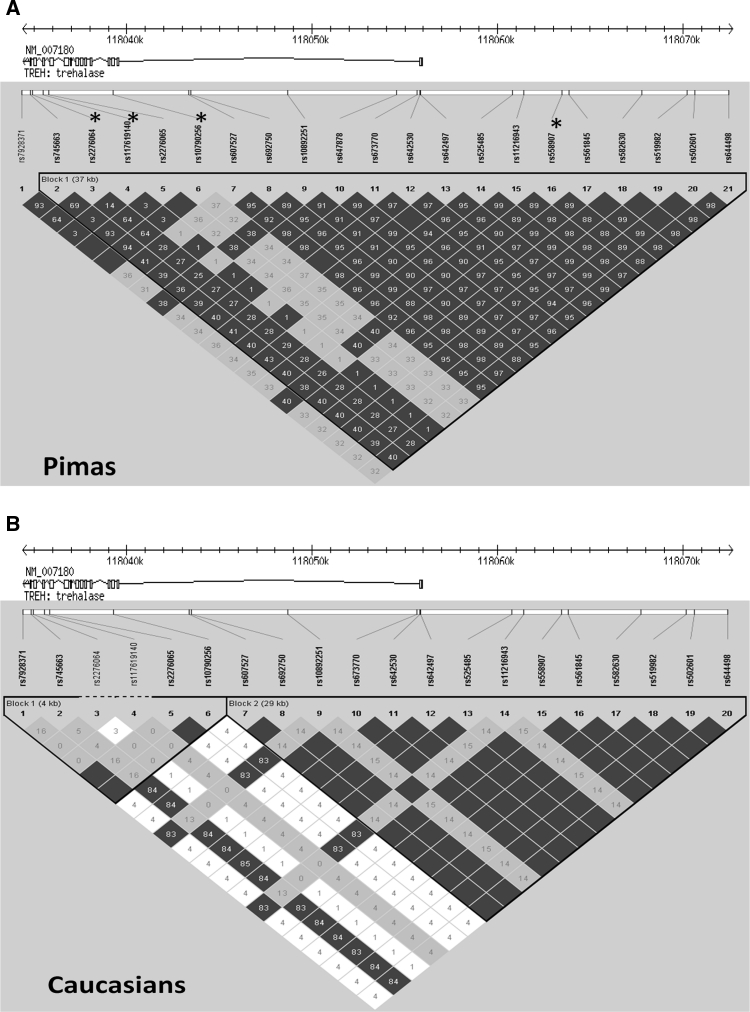
Relative positions of 21 SNPs in and near the *TREH* locus (chr11: 118034337-118072696, NCBI B36) and pair-wise linkage disequilibrium among these SNPs genotyped in 828 Pima Indians (**a**) and 96 Caucasians (**b**). Haplotype block was determined using default confidence interval algorithm implemented in Haploview 4.2. A block was created if 95 % of informative comparisons were “strong LD”, ignoring markers with minor allele frequency <0.05. LD (D’) is displayed as the confidence bounds of *color* scheme where *dark gray* represents “strong evidence of LD”, *light gray* represents “uninformative” and *white* represents “strong recombinant”. The value in the box indicates *r* ^2^. Four tag SNPs indicated by *asterisk* were selected based on *r* ^2^ ≥ 0.8 rs647878 is monomorphic in Caucasians

To search for potentially functional variants in the *TREH* gene, 16 exons and ~2 kb of the upstream region of the *TREH* gene were sequenced in 39 Pima Indians. Four common coding variants (all had a minor allele frequency ≥0.28) were identified: rs10790256 (Lys52Lys), rs2276065 (Thr389Ala), rs2276064 (Arg486Trp) and rs7928371 (Gly541Gly). Recently, whole exome sequence data have been obtained in 177 full-heritage Pima Indians and no additional coding variants in the *TREH* gene were identified in this larger sample. Whole genome sequence data are also being generated, and among 30 genomes currently available, two additional rare coding variants were identified: rs200479357 (Val518Ile) and a novel Leu367Leu (G/T). Each SNP was identified in one heterozygous subject. These rare SNPs were omitted from further study. Sequencing in the 39 subjects also identified eight variants that mapped in the intron/exon junctions or promoter regions: *TREH*-E2B (C/T, +83 bp in intron4, novel), rs673770, rs642497, rs647878, rs10892251, rs692750, rs117619140 and rs745663. To provide a denser coverage of information across this gene, ten additional SNPs (rs607527, rs642530, rs525485, rs11216943, rs558907, rs561845, rs582630, rs519982, rs502601, rs644498), positioned in intron 1 and upstream flanking regions (up to −17 kb) of the *TREH* gene, were selected to determine the pattern of linkage disequilibrium. These SNPs were selected from either our initial systematic association mapping at an average inter-SNP distance of <10 kb across this region, or SNPs from the Affymetrix one million SNP array which had previously been used for genome-wide association studies in Pima Indians (Malhotra et al. 2011). The pattern of LD across the *TREH* locus was evaluated by genotyping these variants in 828 Pima Indians (Fig. 4a) and 96 healthy Caucasian samples (commercially available from Sigma-Aldrich) (Fig. 4b). Among 21 SNPs (the novel intronic variant *TREH*-E2B had a minor allele frequency <0.01 and was therefore omitted), four tag SNPs were selected (using *r* ^2^ >0.8 to indicate redundancy): rs2276064 (Trp486Arg), rs117619140 (intron11), rs10790256 (Lys52Lys, captured rs7928371, rs745663, rs2276065) and rs558907 (a promoter SNP, captured all intron 1 and promoter SNPs: rs607527, rs692750, rs10892251, rs647878, rs673770, rs642530, rs642497, rs525485, rs11216943, rs561845, rs582630, rs519982 rs502601 and rs644498. Recent whole genome sequence data of 30 Pima Indians showed that these four tag SNPs captured all 104 common SNPs (minor allele frequency ≥0.1) in the *TREH* gene (*r* ^2^ > 0.8, Supplemental Figure 1A). These four SNPs also served as tags for Caucasian samples.

### Association of tag SNPs with trehalase activity

The four tag SNPs were genotyped in the 570 Pima Indians who had measures of plasma trehalase activity. Table 1 shows the mean trehalase activity (enzyme units) by genotype. Trehalase enzyme activity was highly associated with three tag SNPs: rs2276064 (Arg486Trp), rs117619140 (intron 11) and rs558907 (promoter) (*p* < 1 × 10^−10^ for all, adjusted for age and sex). The fourth tag SNP rs10790256 (Lys52Lys) was not by itself significantly associated with trehalase activity. When SNPs were tested as to whether the association resulted in a significant reduction in the linkage signal to trehalase activity on chromosome 11q23, the amino acid substitution rs2276064 (Arg486Trp) and rs117619140 (intron 11) produced a statistically significant reduction in the variance attributed to the linked locus (Table 1).

**Table 1:** Associations of 4 tag SNPs in the *TREH* gene with trehalase activity in 570 non-diabetic Pima Indians along with effect on the linkage analysis

Tag SNP	Major/minor allele	Trehalase enzyme activity (Mean ± SE)				Percent variance explained		Linkage analyses		
		Major/major (*n*)	Major/minor (*n*)	Minor/minor (*n*)	*p* value*	Total	Linked locus	LOD_una_	LOD_adj_	*p* value^**†**^
Rs2276064	Trp/Arg	10.83 ± 0.50 (178)	20.50 ± 0.93 (264)	29.30 ± 3.36 (60)	3.48 × 10^−14^	11.0	28.8	5.85	3.65	8.99 × 10^−5^
Rs117619140	G/T	14.68 ± 0.55 (427)	35.14 ± 2.14 (72)	80.27 ± 23.02 (4)	1.41 × 10^−23^	21.3	32.3	5.79	4.44	0.0089
Rs10790256	G/A	17.43 ± 1.00 (255)	18.82 ± 1.13 (217)	19.06 ± 1.82 (40)	0.1291	0.1	2.3	6.24	5.95	0.1050
Rs558907	G/A	20.43 ± 0.84 (362)	12.49 ± 0.85 (141)	4.16 ± 0.77 (10)	2.16 × 10^−11^	9.4	4.0	5.51	5.73	0.1957

Trehalase activity (the enzyme unit) for each genotype is presented as mean ± SE. *p* values are adjusted for age, sex and family membership. Percent variance explained is the percentage of variation in plasma trehalase activity accounted for by association with the SNP (determined by a linear mixed model, after adjustment for age and sex); this percentage is given for the total variance and for the variance attributed to the linked locus

*LOD*_*una*_ LOD score for linkage of trehalase activity (at the peak location of 131 cM) without adjustment for association with the SNP (due to missing genotypic data, this may differ from the overall LOD score), *LOD*_*adj*_ corresponding LOD score with adjustment for the SNP association

* *p* value for the null hypothesis of no association between SNP genotypes and trehalase activity

^†^ *p* value for the null hypothesis that the association explains none of the variance at the linked locus; a scaled normalizing transformation of the ranks was taken in this analysis to improve numerical convergence of the model (Hanson and Knowler 2008)

**Fig. 5:**
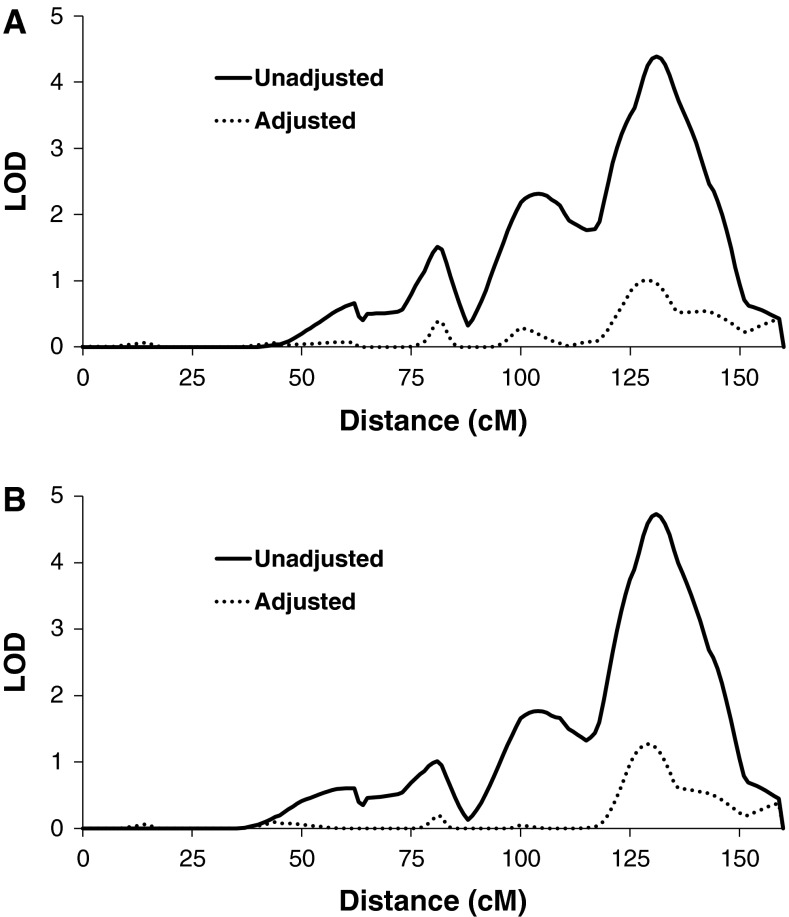
Linkage of trehalase activity adjusted for rs2276064, rs117619140, rs10790256 and rs558907 (**a**); and adjusted for rs2276064 and rs558907 (**b**)

To determine the extent to which multiple variants might contribute to association and linkage with plasma trehalase activity, conditional analyses were conducted in a step-wise fashion. The variant rs117619140 had the strongest association, and when it was included in the model, the SNP with the strongest conditional association among the remaining SNPs was rs558907 (*p* = 3.3 × 10^−8^). When both rs117619140 and rs558907 were included in the model, rs2276064 had the strongest conditional association (*p* = 5.0 × 10^−36^), and when rs117619140, rs558907 and rs2276064 were all included, there was an additional contribution of rs10790256 (*p* = 2.9 × 10^−7^). Thus, together all four tag SNPs are associated with plasma trehalase activity. Likewise, conditional on the effect of rs2276064, there was a significant reduction in the linkage when rs558907 was added to the model (*p* = 0.0035). Conditional on the effects of rs2276064 and rs558907, neither rs117619140 nor rs10790256 produced a statistically significant reduction in the linkage variance (*p* > 0.05). When analyzed together, the four tag SNPs explained a large portion of the linkage signal (Fig. 5a). Association with the four tag SNPs together accounted for 51 % of the total variance in plasma trehalase activity and 79 % of the variance attributed to the linked locus. The two SNPs selected due to a significant reduction in the linkage signal (rs2276064 and rs558907) also resulted in a strong reduction in the linkage signal (Fig. 5b); together these two SNPs explained 47 % of the total variance and 70 % of the variance attributed to the linked locus.

### Association of tag SNPs with T2D

The association with T2D is shown for each of the four tag SNPs in Table 2. Among the 828 individuals who had been included in the linkage study for T2D, tag SNPs rs2276064 and rs558907 were associated with T2D (Table 2, respective odds ratios 1.37 per copy of the T allele, *p* = 0.037, and 1.93 per copy of the G allele, *p* = 0.0024 adjusted for age, sex, birth year and heritage). In genotyping of additional individuals from the population who had not participated in the linkage study, tag SNP rs558907 was associated with T2D in both full-heritage Pima Indians (Table 2, OR 1.27, *p* = 0.0291, *n* = 2,942), and in those individuals who were not full-heritage Pimas (Table 2, OR 1.21, *p* = 0.0321, *n* = 3,897). The strongest association with T2D was seen in a combined analysis of all subjects (Table 2, OR 1.27, *p* = 1.6 × 10^−4^, *n* = 7,667). None of these SNPs were consistently associated with body mass index (Supplemental Table 1).

**Table 2:** Associations of 4 tag SNPs in the *TREH* gene with T2D in Pima Indians

Tag SNP	Risk/Non	Participants in linkage study			Participants not included in linkage study							
		All linkage study (*n* = 828)			Full-heritage pima (*n* = 2,942)			“Mixed” heritage pima (*n* = 3,897)			All combined (*n* = 7,667)	
		RAF	OR (95 % CI)	*p* value	RAF	OR (95 % CI)	*p* value	RAF	OR (95 % CI)	*p* value	OR (95 % CI)	*p* value
Rs2276064 Trp486Arg	T/C	0.63	1.37 (1.02–1.85)	0.0372	0.63	1.00 (0.88–1.14)	0.9975	0.46	1.13 (0.98–1.32)	0.0936	1.09 (1.00–1.22)	0.0738
Rs117619140 intron 11	T/G	0.08	1.10 (0.64–1.89)	0.7255	0.08	1.11 (0.88–1.41)	0.3606	0.13	0.88 (0.71–1.09)	0.2557	0.98 (0.85–1.14)	0.8030
Rs10790256 Lys52Lys	G/A	0.72	1.39 (1.00–1.92)	0.0518	0.73	1.04 (0.90–1.21)	0.5542	0.74	1.00 (0.86–1.17)	0.9906	1.05 (0.95–1.16)	0.3458
Rs558907 promoter	G/A	0.87	1.94 (1.26–2.96)	0.0024	0.89	1.27 (1.02–1.58)	0.0293	0.76	1.21 (1.01–1.43)	0.0333	1.27 (1.12–1.44)	1.6 × 10^−4^

Analysis for “all” is a combined analysis of individuals in the linkage study, full-heritage Pima Indians and “mixed-heritage” subjects. The risk allele (given first) is defined as the allele with a higher risk of diabetes in the linkage study; odds ratios (ORs) are given per copy of this allele. RAF is the frequency of the risk allele

### Association of haplotypes with trehalase activity and T2D

**Fig. 6:**
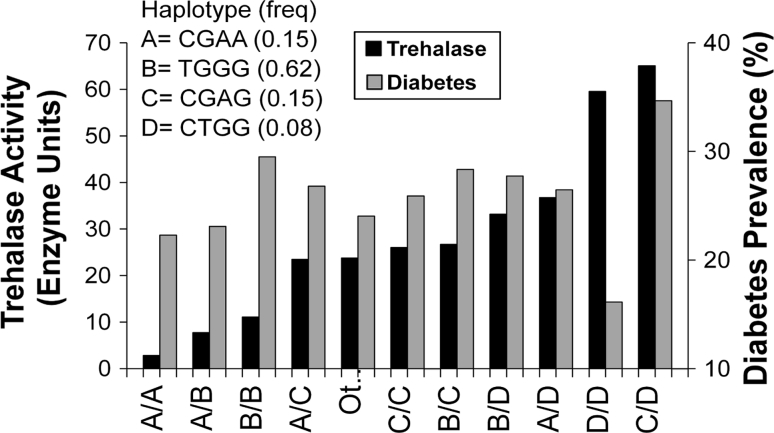
Trehalase activity and diabetes in Pima Indians by haplotypes in *TREH*. The haplotypes, A, B, C, and D are defined by the variants in the following order: rs2276064, rs117619140, rs10790256, rs558907. Haplotype frequencies for those participants in the linkage study are indicated in parentheses

To further explore the genetic associations, haplotype analyses were conducted. Among the four tag SNPs, there were four common (frequency >0.05) haplotypes. Analyses of trehalase activity showed that haplotype “A” (CGAA), which contained the C allele at rs2276064, the G allele at rs117619140, the A allele at rs10790256 and the A allele at rs558907, tended to have the lowest activity (Fig. 6). Those with haplotype “D” (CTGG), consisting of the C allele at rs2276064, the T allele at rs117619140, the G allele at r10790256, and the G allele at rs558907, tended to have the highest activity. The other haplotypes were intermediate between these two, with the “B” haplotype (TGGG) having lower activity than the “C” haplotype (CGAG). When diabetes was analyzed according to the same haplotypes, the A haplotype which was associated with low trehalase activity, was also associated with a low prevalence of T2D, particularly when homozygous, and the combination of C and D had the highest prevalence with diabetes and the highest trehalase activity. However, there was otherwise little correspondence between the effect of a haplotype on trehalase activity and diabetes risk. When the haplotype combinations were ordered according to their association with the level of trehalase activity, there was little association with T2D (*p* for trend = 0.34, Fig. 6).

### TREH activity and T2D prediction

Although all individuals were nondiabetic at the examination for which trehalase activity was measured, 320 had been subsequently examined in the longitudinal study and 214 had developed diabetes. Among these individuals trehalase activity did not significantly predict development of diabetes (hazard ratio 0.93 per SD increase in trehalase activity, 95 % CI 0.81–1.07, *p* = 0.29). In an additional small nested case–control study comparing 48 individuals who initially had normal glucose tolerance, but who subsequently developed diabetes, with matched controls who remained free of diabetes in follow-up (Lindsay et al. 2002, Krakoff et al. 2003), baseline trehalase activity also did not predict diabetes (hazard ratio 1.18, 95 % CI 0.75–1.85, *p* = 0.48).

## Discussion

Plasma trehalase activity is strongly linked (LOD = 7.0) to a region near the *TREH* locus on chromosome 11q23 in 570 non-diabetic Pima Indians. Genetic variants in or near the *TREH* locus are strongly associated with trehalase activity and account for a large portion of the linkage signal. One of these variants, rs558907 is also reproducibly associated with T2D in three non-overlapping samples of Pima Indians.

That genetic polymorphisms influence plasma trehalase activity has been suggested decades ago in Eze (1989). In his report, a bimodal distribution of activity was observed in 30 normal Nigerian subjects, suggesting a potential underlying genetic influence. We identified four tag SNPs that are associated with the overall trehalase activity in plasma of Pima Indians. These associations are strong in that most achieve the statistical threshold typically required for genome-wide significance (*p* < 7.2 × 10^−8^) (Dudbridge and Gusnanto 2008). Together, these SNPs explain 51 % of the variance in trehalase activity and 79 % of the variance attributed to the linked locus, thus accounting in large part for the observed linkage signal. This suggests that these variants are in linkage disequilibrium with functional variants that influence trehalase activity. It is noteworthy that in the initial analyses of the effect of *TREH* variants on trehalase activity, some of the SNPs had a negligible effect on the linkage signal, despite strong evidence for association (e.g., rs558907). However, the association with rs558907 produced a substantial and significant effect on the linkage signal when adjusted for the effect of rs2276064. This may occur because none of the SNPs individually accounts for the entire linkage signal and, in this situation, adjustment for a variant that partially accounts for the signal may actually enhance power to detect residual linkage. More broadly, this suggests that additional susceptibility variants should be sought in regions implicated by linkage analysis for complex traits and in which strong associations have been observed that do little to explain the linkage signal.

Previous studies have linked plasma/serum trehalase activity to T2D in humans (Eze 1989; Isichei and Gorecki 1993). At present, the mechanism of the association between trehalase activity and T2D is unknown. Plasma trehalase activity was higher in subjects with than without T2D in both Nigerian and British populations (Eze 1989). Higher serum trehalase activity was also observed in diabetic subjects with glycosuria compared to diabetic subjects without glycosuria (Isichei and Gorecki 1993). In mice, trehalase activity was elevated in both alloxan-induced and genetic (Ob/Ob, Db/Db) diabetic mice (Baumann et al. 1981; Ramaswamy and Flint 1980). However, it is not clear whether elevated trehalase activity observed in these studies is the effect of T2D, or if people with higher trehalase activity, perhaps due to linked genes, are more prone to develop T2D. Moreover, no significant correlation was observed between plasma/serum trehalase activity and blood glucose levels in these studies or in the present study (data not shown). Among 241 non-diabetic, full-heritage Pima Indians who had undergone detailed metabolic testing and also had serum collected, trehalase activity was associated with basal carbohydrate oxidation (*p* = 0.03, adjusted for age, sex and percentage body fat) and carbohydrate oxidation under low- and high-dose insulin stimulations (adjusted *p* = 0.007 and 0.009, respectively). Higher trehalase activity was associated with lower carbohydrate oxidation. However, it remains to be determined if trehalase is involved in regulation of blood glucose level and/or glucose oxidation, if trehalase activity changes secondary to these processes or if both are influenced by correlated factors. A recent study by Boyd et al. (2009) has indicated that the *TREH* gene expression was directly regulated by the transcriptional factor HNF4α via binding of the *TREH* promoter in human intestinal epithelial cells. HNF4α has been extensively studied in hepatocytes and pancreatic β-cells for its role in development of MODY (maturity onset diabetes of the young) and T2D. However, in the present study we did not find that plasma trehalase activity measured at a non-diabetic stage directly predicts the development of T2D in Pima Indians, although our sample size for this analysis (*n* = 320) may have low statistical power to detect a modest predictive effect of trehalase activity on T2D as indicated by the confidence interval of the hazard ratio (95 % CI 0.81–1.07).

In the present study, a tag SNP in the *TREH* gene, rs558907, was associated with T2D in Pima Indians. The association with T2D was observed initially in individuals who participated in a linkage study for T2D, and consistent results were observed in other members of the population, both those of full Pima heritage and those of mixed heritage. The level of statistical significance (*p* = 1.6 × 10^−4^), however, does not achieve that typically required for genome-wide significance (Dudbridge and Gusnanto 2008), so it remains possible that the diabetes association is spurious. However, it should be noted that in all of the genetic studies that have been conducted in Pima Indians to date, which include genome-wide association studies using both the 100 K and one Million Affymetrix SNP chips (Malhotra et al. 2011; Hanson et al. 2007 and unpublished), the association in *TREH* remains among the top 15 strongest associations with T2D for variants genotyped in or near ~1800 genes. It is, however, likely that the functional variant for the T2D association may not be rs558907 or, given the extent of linkage disequilibrium in the region, in *TREH* itself. The flanking region of the *TREH* gene is in a large haplotype block and contains several additional known and predicted genes including *PHLDB1* and *DDX6* (Supplemental Figure 1B). Because of the high degree of LD between variants in the *TREH* locus and nearby loci, localization of any diabetes association may be difficult.

Ultimately, replication in other populations with lower LD across this region may be required to determine whether the association between *TREH* variants and T2D is spurious or real. At present, there are few data, except from Caucasian populations. From 13 *TREH* SNPs (rs7928371, rs745663, rs10790256, rs607527, rs692750, rs673770, rs642530, rs525485, rs11216943, rs561845, rs582630, rs519982 and rs644498) directly genotyped or imputed in the large Caucasian DIAGRAM study, meta-analysis has shown no association between these SNPs with T2D (Zeggini et al. 2008). In Caucasians, these 13 SNPs were captured by two tag SNPs, rs10790256 and rs558907. Although rs558907 was not genotyped in DIAGRAM study, a proxy, rs692750 (*r* ^2^ = 1 with rs558907) was genotyped in DIAGRAM study, and not associated with T2D in Caucasians (*p* = 0.79). Further replication studies in other, particularly non-Caucasian, populations are required to determine the relative contribution of this gene to T2D susceptibility.

Our results indicate that SNPs in *TREH* strongly influence trehalase activity. However, the exact functional variant(s) which contribute to the trehalase activity have not been identified. The programs “SIFT” and “PolyPhen”, which bioinformatically predict the effects of coding variants, both predict a “tolerated” effect of Arg486Trp (rs2276064) on protein function. This does not necessarily preclude an important effect of this variant on plasma trehalase activity, but, alternatively, intronic or promoter variants could be involved in the regulation of *TREH* expression. Variant analysis by Ingenuity Pathway Analysis predicts the loss of promoter function of rs642530, rs673770 and rs36077162 which are in complete LD with rs558907 (*r* ^2^ = 1). Therefore, future functional studies are required to further define the causative variant(s) in this gene. Although our association analyses showed that SNPs in and/or near the *TREH* gene were associated with T2D, it remains unclear whether the diabetes causative variant(s) are within the *TREH* gene or a nearby gene. Furthermore, since there was little correspondence between the effect of *TREH* haplotypes on trehalase activity and diabetes risk, and little evidence that trehalase activity itself predicts T2D, it is likely that the associations between *TREH* variants and T2D are mediated by a different mechanism than the effects on trehalase activity.

## Electronic supplementary material

Below is the link to the electronic supplementary material.

Supplementary material 1 (DOCX 706 kb)
