# Supplementary material for: Identification of genetic variation that determines human trehalase activity and its association with type 2 diabetes
Source: Hum Genet. 2013 Mar 7;132(6):697–707. doi: 10.1007/s00439-013-1278-3 (PMC3654185; doi:10.1007/s00439-013-1278-3)
Supplement: Supplementary file 1 — Supplementary material 1 (DOCX 706 kb) [file 439_2013_1278_MOESM1_ESM.docx]

**Supplemental Fig. 1**

**Supplemental Figure 1A: Pair-wise linkage disequilibrium among 104 SNPs (AF ≥ 0.1) identified from whole genome sequencing of 30 Pima Indians in and near the *TREH* locus (chr11:118034337-118072696, NCBI B36)**. LD (D’) is displayed as the confidence bounds color scheme where dark gray represents “strong evidence of LD”, light gray represents “uninformative” and white represents “strong recombinant”.

**A.**

**
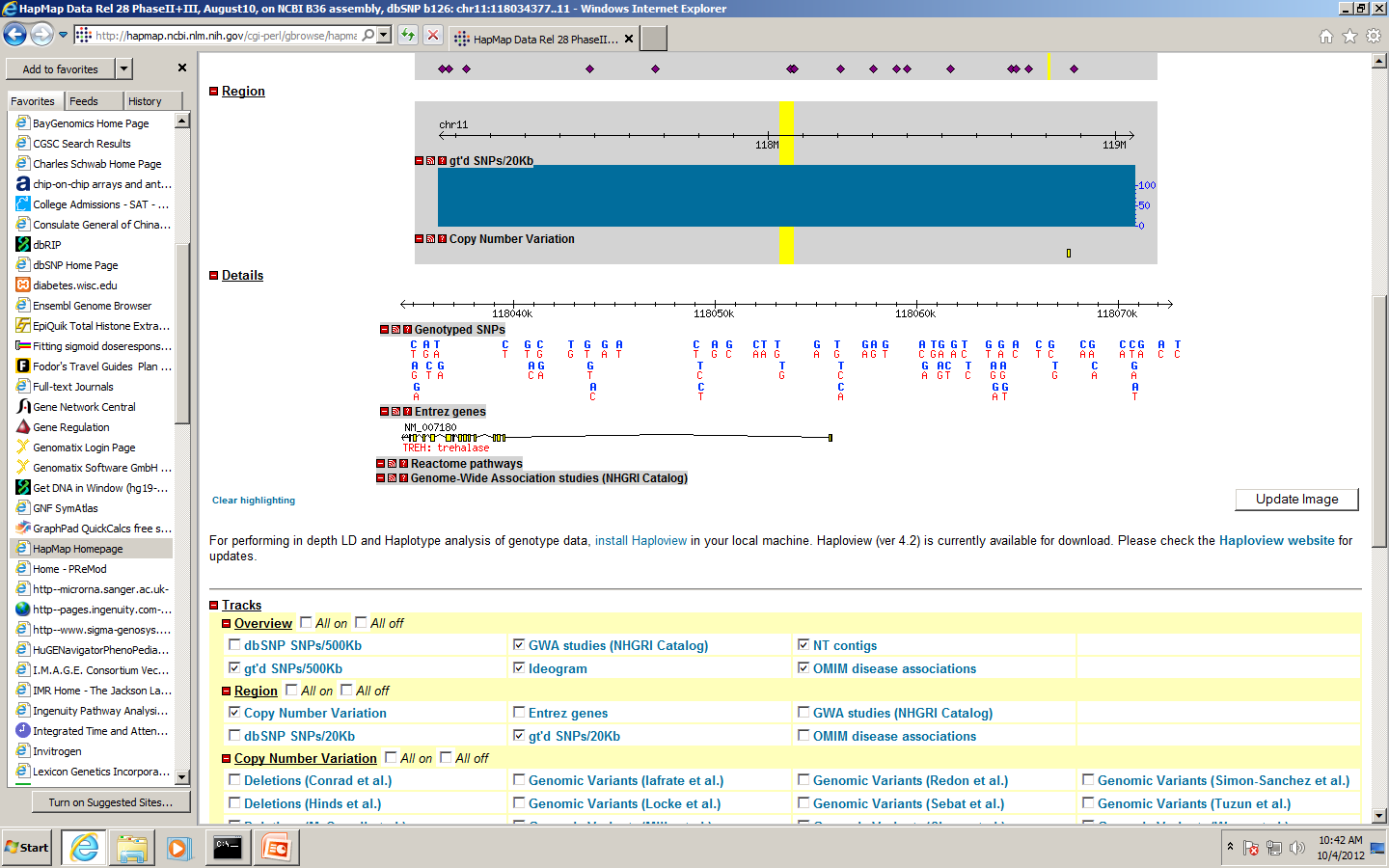

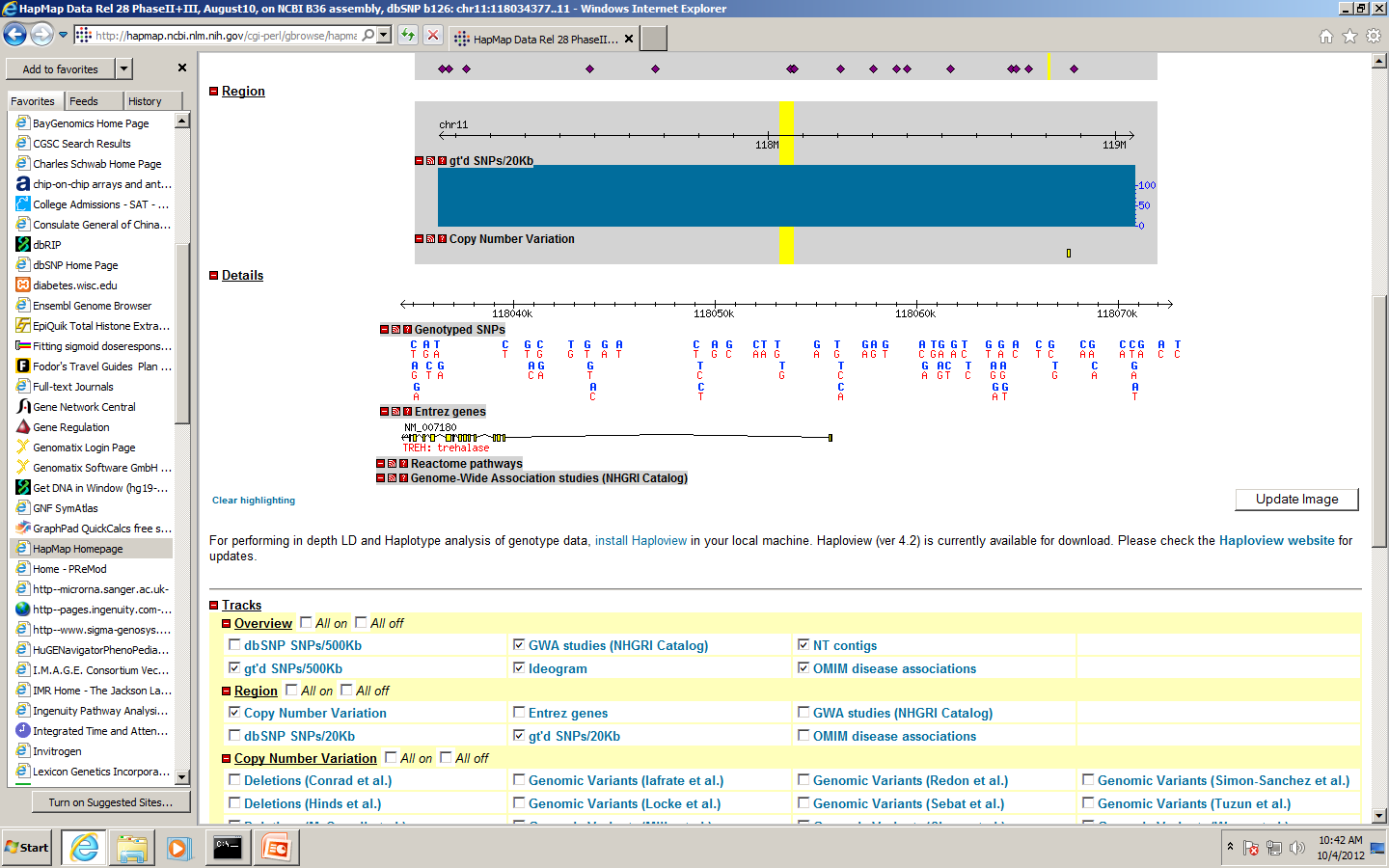
**


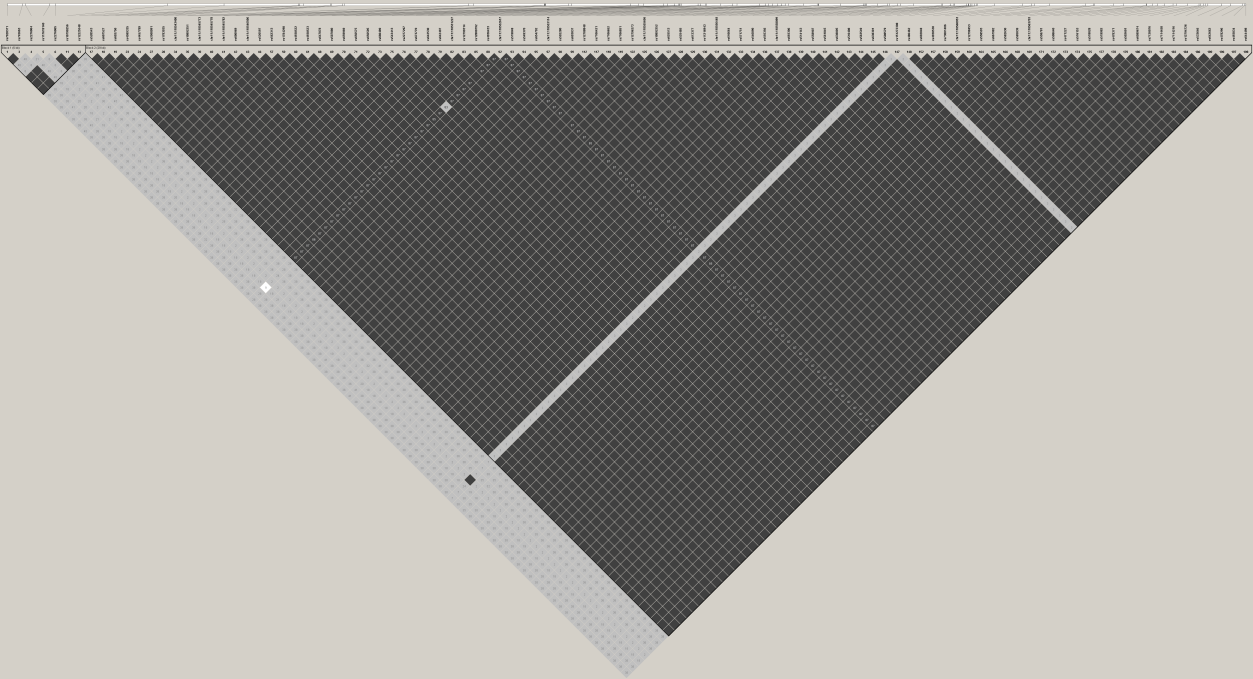


**Supplemental Figure 1B: Pair-wise linkage disequilibrium among 67 SNPs across ~ 426 kb region encompassing *TREH, PHLDB1* and *DDX6* (chr11:117825763-118251979, NCBI B36) in 828 Pima Indians who participated in the linkage study for T2D.** LD (D’) is displayed as the confidence bounds of color scheme where dark gray represents “strong evidence of LD”, light gray represents “uninformative” and white represents “strong recombinant”. SNPs with minor allele frequency <0.05 were omitted.

**B.**

**Supplemental Table 1. Associations of *TREH* SNPs with body mass index in Pima Indians**. BMI is the maximum value observed in the longitudinal study from all examinations after age 15 years. The risk allele (given first) is defined as the allele with a higher risk of diabetes in the linkage study; the regression coefficient (beta) represents the difference in the logarithm of BMI (in SD units) per copy of this allele. RAF is the frequency of the risk allele.

|  |  | Participants in Linkage Study | | | Participants not Included in Linkage Study | | | | | | | |
| --- | --- | --- | --- | --- | --- | --- | --- | --- | --- | --- | --- | --- |
| Tag SNP | Risk/  Non | All Linkage Study (n=822) | | | Full Heritage Pima (n=2732) | | | Not Full Heritage Pima (n=3232) | | | All Combined (n=6786) | |
|  |  | RAF | beta  (95% CI) | P-value | RAF | beta  (95% CI) | P value | RAF | beta  (95% CI) | P value | beta  (95% CI) | P value |
| rs2276064  Trp486Arg | T/C | 0.63 | 0.07  (-0.05, 0.19) | 0.2324 | 0.63 | -0.03  (-0.08,-0.02) | 0.2960 | 0.46 | 0.05  (-0.01, 0.10) | 0.1042 | 0.02  (-0.02, 0.05) | 0.3987 |
| TREH-E2I2  Intron 11 | T/G | 0.08 | 0.09  (-0.13, 0.34) | 0.4693 | 0.08 | 0.05  (-0.15, 0.04) | 0.2380 | 0.13 | -0.06  (-0.13, 0.02) | 0.1240 | 0.01  (-0.05, 0.06) | 0.8482 |
| rs10790256  Lys52Lys | G/A | 0.72 | 0.11  (0.00-0.22) | 0.0441 | 0.73 | -0.02  (-0.08-0.04) | 0.4710 | 0.74 | -0.01  (-0.07-0.05) | 0.8409 | 0.00  (-0.04, 0.04) | 0.9868 |
| rs558907  Promoter | G/A | 0.87 | 0.12  (-0.09, 0.29) | 0.1865 | 0.90 | -0.02  (-0.11, 0.06) | 0.5991 | 0.76 | 0.04  (-0.03, 0.10) | 0.2792 | 0.03  (-0.02, 0.08) | 0.2190 |
